# Pragmatic cluster randomised trial of a free telephone-based health coaching program to support women in managing weight gain during pregnancy: the Get Healthy in Pregnancy Trial

**DOI:** 10.1186/s12913-016-1704-z

**Published:** 2016-08-30

**Authors:** Vanessa Clements, Kit Leung, Santosh Khanal, Jane Raymond, Michelle Maxwell, Chris Rissel

**Affiliations:** 1 NSW Kids and Families, Sydney, Australia; 2 Public Health Officer Training Program, NSW Ministry of Health, Sydney, Australia; 3 NSW Office of Preventive Health, Sydney, Australia; 4 Centre for Population Health, NSW Ministry of Health, Sydney, Australia

**Keywords:** Health promotion, Health coaching, Gestational weight gain, Obesity, Maternal obesity

## Abstract

**Background:**

Excessive gestational weight gain can result in poor maternal and child health outcomes. Estimates from single studies indicate the prevalence of excessive gestational weight gain in Australia could lie between 38 and 67 %. The risk of excessive weight gain can be reduced through healthy eating and exercise. We describe the rationale and methods of the Get Healthy in Pregnancy Service, a trial service which aims to support women in achieving appropriate gestational weight gain through an existing telephone-based health coaching service.

**Methods/Design:**

This study aims to compare the effectiveness of a telephone-based health coaching program versus provision of information only in supporting pregnant women to achieve appropriate gestational weight gain. A pragmatic stratified clustered randomised controlled trial will be conducted with 710 women who present to 5 hospitals for their first antenatal appointment during the recruitment period (6–8 months), have a pre-pregnancy body mass index (BMI) ≥ 18.50 (healthy weight or above), are 18 years and over, singleton gestation, English speaking, have no pre-existing medical conditions that may limit their ability to exercise or require a restricted diet and are 18 weeks or less gestation. Hospitals will be randomised into one of two intervention models: a) information only; or b) information plus 10 telephone-based health coaching sessions with a university qualified coach. Both interventions will set a weight-range target with pregnant women. The women attending antenatal clinics at participating hospitals will be screened at their initial hospital appointment to assess their eligibility. Women recruited to the trial will have a number of measures recorded including anthropometrics (self-reported height and weight) and dietary and physical activity scores during and following pregnancy. These measurements will be collected at baseline (prior to 18 weeks gestation), 36 weeks gestation and 12 months post-birth.

**Discussion:**

This study responds to a need for an effective intervention that targets excessive gestational weight gain at a population level. This study investigates the potential for an innovative intervention combining two existing services; a free state-wide telephone-based health coaching service and hospital-based antenatal care to support pregnant women to achieve healthy weight gain during pregnancy. The use of existing services provides the potential for immediate post-study implementation. While the impacts of telephone-based lifestyle programmes have been tested in a number of settings, there are few studies which evaluate the effectiveness and acceptability of telephone support in achieving healthy gestational weight gain in association with routine antenatal care.

**Trial registration:**

ACTRN12615000397516 (Registration date: 26 June 2014, retrospectively registered).

**Electronic supplementary material:**

The online version of this article (doi:10.1186/s12913-016-1704-z) contains supplementary material, which is available to authorized users.

## Background

Excessive gestational weight gain (EGWG) is associated with poor maternal and infant health outcomes, including an increased risk for gestational hypertension, gestational diabetes, caesarean section, low apgar scores and infants who are born large for gestational age [1–4]. There is also an increased likelihood of postpartum obesity in both mothers [2] and their children [5], which in turn leads to an increased risk of chronic disease later in life [6]. Risk factors for EGWG include a high pre-pregnancy BMI, with some studies showing that women who are overweight or obese prior to pregnancy are 2–6 times more likely than women with a normal BMI to be at risk of EGWG [7, 8]. EGWG in women of all pre-pregnancy BMIs can lead to a progressive weight gain which is retained over a woman’s reproductive years [9].

The Institute of Medicine (IOM) gestational weight guidelines [10] were updated in 2009 and are the most frequently utilised internationally. The guidelines provide ranges of recommended weight gain for specific pre-pregnancy BMI categories, based on the least risk of adverse perinatal outcomes [11]. It has been suggested that the gestational weight gain for most women in reality exceeds the IOM recommendations [11, 12], and in Australia, the prevalence of EGWG has been reported as being between 38 and 67 % [13, 14]. The first national guidelines for antenatal care in Australia were published in August 2013, recommending that clinicians give women advice about appropriate weight gain during pregnancy in relation to their pre-pregnancy BMI [15].

Diet and exercise are modifiable risk factors for EGWG, and a number of interventions have been trialled to reduce EGWG by changing dietary and physical activity behaviours. Evidence to date however is mixed with some interventions either not resulting in a significant decrease in EGWG or only proving effective in certain groups, such as women with a normal pre-pregnancy BMI [11, 16, 17]. Changes in diet and physical activity can result in lower weight gain but what is not known is the mechanism by which this is achieved.

Most reported interventions are resource-intensive in terms of face to face specialist professional support and therefore unsustainable in the long term. Simple strategies such as giving women accurate advice regarding a target gestational weight gain [18], and encouraging women to self-weigh [19] appear to be effective in some groups. As in the non-pregnant population, intensive initiatives that provide regular reminders appear to produce the most effective results [20]. It seems that these do not have to be delivered directly by clinicians; Soltani et al. [21] utilised text messaging to deliver simple regular reminders with positive results, although the study numbers were too small for statistical analysis.

A recent study by Dodd et al. [22] employing a combined telephone and face to face intervention for overweight and obese pregnant women did not find any improvements in maternal and birth outcomes compared with standard care, but their study sample was skewed towards women from high socio-economic backgrounds who are more likely to have a higher baseline for health behaviours than the general population [23]. The study also did not provide women with a target gestational weight gain or evaluate the acceptability of the service.

The process of telephone-based health coaching, delivered by qualified dietitians and exercise physiologists unrelated to the client’s clinical care, is a relatively new concept for maternity care providers, although well established for patients with chronic disease [24]. It is designed to support clients through regular contact to achieve a healthier lifestyle through the use of behaviour change theory, thereby also supporting the work of time-poor clinicians. A free telephone-based health coaching, the Get Healthy Information and Coaching Service (GHS), has been available to all adults over 18 years of age in New South Wales (NSW), Australia since 2009. People using this service can opt to receive information only or enrol in a six month coaching program. The program consists of 10 individually tailored calls from university qualified coaches, aimed at making sustained improvements in healthy eating, physical activity and achieving or maintaining a healthy weight. Extensive evidence exists for the effectiveness of telephone-based lifestyle programs and evaluation of the GHS itself shows that participants who complete the 6 month coaching program lose an average of 3.9 kgs and reduce their waist circumference by 5.0 cms. [25] The GHS has a considerable proportion of participants who are female (73 %) and reside outside of major cities (40 %) as well as being from lower socio-economic backgrounds [26].

### The NSW Get Healthy in Pregnancy Service (GHiP)

A telephone-based service for pregnant women of New South Wales (NSW), Australia, Get Healthy in Pregnancy (GHiP), was developed in collaboration between the NSW Office of Preventive Health and NSW Kids and Families to support women to achieve appropriate gestational weight gain through health coaching. GHiP was developed as an enhancement to the GHS. Development of GHiP has been informed by evaluations of other interventions aimed at reducing EGWG with the input of an Expert Advisory Panel including midwives, obstetricians, dietitians and antenatal educators from across NSW. Pregnant women have suggested in a qualitative study in the UK that they would prefer a healthy eating service that is integrated into routine care and provides information early in their pregnancy [27]. Two potential models for the GHIP integrated with hospital antenatal care have been developed and will be compared as the two arms in this study:

- *Information only* – introduction of gestational weight gain recommendations by midwife at their initial hospital antenatal appointment, evidence-based factsheets and an information booklet on healthy eating, exercise and weight gain during pregnancy in line with Australian guidelines [28–30], and a single health coaching call; and
- *Health coaching* – introduction of gestational weight gain recommendations by midwife at their initial hospital antenatal appointment, the same factsheets and information booklet as the information only group, plus a journey booklet to record progress and up to 10 health coaching calls (8 during pregnancy, 2 post pregnancy).

Both service models commence between 12 and 22 weeks gestation. Participants receiving health coaching are offered coaching calls up until 36 weeks gestation and receive two coaching calls at 10 and 14 weeks post-delivery.

## Methods/Design

### Aims and objectives

This study will compare the effectiveness of a telephone-based health coaching program versus provision of information alone in supporting pregnant women to achieve appropriate gestational weight gain. Pregnant women will be stratified into pre-pregnancy healthy weight and overweight/obese groups. Given the evidence around issues related to excessive weight gain in pregnancy, a pure control group was not included in the study as it was considered inappropriate to not provide information that could be beneficial to maternal and neonatal health.

The hypothesis of the study is that, pregnant women enrolled in the coaching program will be more likely to achieve a healthy gestational weight gain during pregnancy and better maintain their pre-pregnancy weight post pregnancy. Weight-gain that is above the IOM guidelines is considered excessive gestational weight-gain.

The primary objectives of the study are to:

i) Compare the differences in gestational weight gain and weight retention at 12 months post birth of participants in the health coaching and information only arms
ii) Understand the acceptability and experiences of telephone-based coaching amongst pregnant women and antenatal service providers.

As secondary objectives, the study will:

iii) Compare changes in diet (fruit, vegetable, fast food and sweetened drink intake)
iv) Assess the predictors of service participation and retention
v) Explore service usage patterns such as length of calls and preferred time
vi) Assess confidence to change
vii) Provide recommendations for program improvement.

### Study design

This study employs a pragmatic stratified cluster randomised design [31] with stratification by healthy weight and overweight/obese pre pregnancy BMI.

**Fig. 1:**
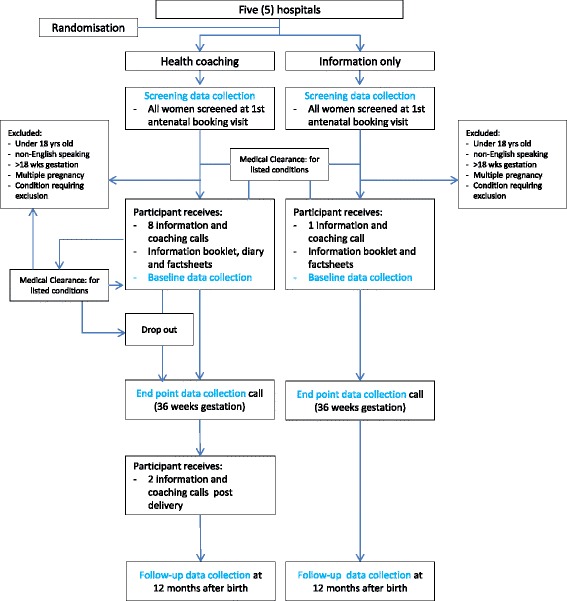
Study flowchart

Pregnant women in the study will be allocated to either the information only or health coaching group based on the hospital where they present for antenatal care (Fig. 1). This design was selected to obtain results that can be generalised with high external validity and utility in the real world setting. In this study, the trial environment and processes will be as close as possible to how the program would be rolled out if effective. A range of hospitals in both metropolitan and rural settings, of different sizes and maternity service delivery levels have been chosen to test the scalability of the intervention across the state.

Randomisation of hospitals, stratified by metropolitan and rural regions, will be undertaken using Microsoft Excel 2010© randomisation generator. Two rural hospitals were combined into one unit for randomisation because they are in the same Local Health District and their aggregated total number of births in 2012 was similar to the total of the other rural hospital in the study. Face-to-face training will be provided, by members of the research team, to the midwives and medical officers at each site to describe the study and the program. The hospitals will develop their own processes to integrate the GHiP referral into the first antenatal appointment for pregnant women. As part of this process, hospitals in both arms will be required to include initial discussion on the importance of healthy gestational weight gain, the setting of a target weight-gain range for the woman’s pregnancy (as per IOM recommendations), screening of the woman for exclusion criteria and recruitment of women following consent.

Health coaches (primarily dieticians and exercise physiologists) delivering GHiP will be provided with a day’s face-to-face training by clinical experts in dietetics and maternity care, in addition to their usual training. A list of online resources to support the pregnant women will also be available to all health coaches. The clinical experts will also be available for further consultation as required.

### Study setting

The study will be conducted in the antenatal clinics of five NSW hospitals of which three are rural (Orange Base, Lismore Base and Dubbo Base) and two metropolitan (Liverpool and Blacktown). Hospitals were recruited through an expression of interest process addressed to the general manager of each site in the first instance. Agreement was also obtained from the midwifery unit manager and obstetric lead of the maternity unit at each hospital to ensure local support prior to inclusion. All hospitals are public hospitals accounting for approximately 10 % of the total births in NSW and the demographic characteristics of women attending the two metropolitan and the three rural hospitals are similar within their categories. The hospitals offer a variety of antenatal care options for women including hospital-based, shared care with general practitioners or via community based midwifery group practices. All of these care models require women to attend the hospital for an initial antenatal appointment which usually occurs after 12 weeks of gestation.

### Recruitment strategies

Pregnant women who are presenting at one of the five participating hospitals will receive a flyer with their initial appointment letter and advised that their midwife will discuss the Get Healthy in Pregnancy service and research trial with them at their first appointment. Midwives during the recruitment period will screen and assess every woman presenting for their first antenatal visit to determine eligibility to be enrolled in the study. Women eligible to participate will be offered the opportunity to participate in the trial and asked for written consent. Women who are unsure whether they participate will be contacted by a research assistant within a week and reoffered the opportunity to participate.

### Participants

Participants will be 710 pregnant women who attend one of five hospitals during the recruitment period and are English speaking, 18 years and over, have a singleton pregnancy, have a gestation of 18 weeks or under and agree to participate (signed consent forms and verbal consent provided at first coaching call).

### Medical conditions

An exclusion list and requirements for medical clearance (Table 1) for women with conditions that may impact their involvement was established to ensure safety of the women participating in the study. The national guidelines for consultation and referral by midwives [32] were used to identify the medical conditions for exclusion or medical clearance. For the purpose of this study, all conditions identified within the guidance as requiring referral to a medical practitioner for specialist care were reviewed by the GHiP Expert Advisory Group with regard to the woman’s ability to be able to safely modify her diet or levels of activity.

**Table 1:** Medical conditions for exclusion from the study or requiring clearance from a medical practitioner

	Pre-existing conditions (at screening)	Conditions developed during the study period
Excluded from the study	Cardiovascular disease: • Arrhythmia/palpitations; murmurs: recurrent, persistent or associated with other symptoms • Cardiac valve disease • Cardiac valve replacement • Cardiomyopathy • Congenital cardiac disease • Ischaemic heart disease • Pulmonary hypertension Endocrine: • Pre-existing Type 1 diabetes • Cystic fibrosis • Phenylketonuria (PKU) Respiratory Disease • Sarcoidosis Severe lung disorder	• Fetal death in utero • Small for dates (<10th centile) • Multiple pregnancy • Preterm birth (<36 weeks) • Rupture of membranes (<36 weeks) Placental indications: • Placental abruption • Placenta accrete • Placenta preavia • Vasa Praevia
Medical clearance required to participate	Cardiovascular disease • Hypertension Neurological • Epilepsy Respiratory disease • Asthma Moderate (women requiring daily bronchodilators and steroid inhalers) • Type 2 diabetes (diet/medication controlled) Skeletal problems • Musculo-skeletal problems Previous obstetric history • Previous pre-term birth • Recurrent miscarriage History of mental health • History of mental health conditions (e.g. eating disorders) Endocrine • Type 2 diabetes (diet/medication controlled) Gastro-intestinal Coeliac Disease	Endocrine • Pre-existing Type 2 diabetes now requiring medication Skeletal problems • Herniated vertebral disc • Symphysis pubis dysfunction Cardiovascular disease Chronic hypertension Obstetric related • Anaemia (Hb <90) • Fetal size small for dates • Vaginal blood loss • Pre-eclampsia • Gestational diabetes – diet/medication controlled Gestational hypertension

For women requiring medical clearance, the participant file will be forwarded to a medical practitioner for review. During the review the medical practitioner will be required to select from three options; clear the woman for full participation in the trial, exclude from the trial, or provide parameters within which the woman can participate (eg. restricting certain forms of physical activity or certain variations to the woman’s diet).

### Sample size

A total of 710 women will be recruited; 177 and 532 pregnant women with a pre-pregnancy BMI of 18.5–24.9 kg/m^2^ (healthy range) and ≥25.0 kg/m^2^ (overweight or obese range) respectively. 248 women will be recruited across the three rural hospitals with 462 recruited at metropolitan hospitals. The higher numbers in metropolitan hospitals is to reflect the larger target population in these areas.

The sample size has been calculated using previous reports of weight gain during pregnancy [10, 13, 33] to detect a difference of 3 kgs in gestational weight gain between intervention and control groups at 80 % power. Attrition rates of 60 % in the coaching arm and 30 % in the information only arm are expected. These rates account for the up to 15 % of women, across both arms, who start the study and go on to develop conditions during pregnancy that exclude them from further participation [34] and, allows for the perception of participant burden in the coaching arm. The relatively high attrition rate is conservative compared with other population based programs.

### The interventions

#### Coaching arm

Women in the coaching arm will be enrolled in a full coaching program comprising of up to 10 calls by university qualified coaches (8 during pregnancy and 2 after birth). Health coaches, using behaviour change informed coaching techniques, will in their first call confirm and discuss the woman’s weight range target according to the IOM guidelines, and develop personally relevant lifestyle change goals and actions for participants to achieve their target weight range. In subsequent coaching calls health coaches will ensure that no conditions have presented in the pregnancy that may require medical clearance or adapted advice, revisit goals and revise them accordingly with the participant, help maintain motivation, discuss strategies for overcoming barriers and prevent relapse of healthy behaviours. Health coaches are guided by a range of national and international guidelines for nutrition, exercise and weight-gain targets for pregnancy. The timing of calls is designed to be flexible based on participant preferences and will generally last between 15 and 25 min. A recommended schedule for the pregnancy coaching calls will be used; three in the first 3 weeks, followed by a call every 2–4 weeks until the end of the pregnancy with two calls post birth.

In addition, participants in the health coaching group will be provided with supportive materials including pregnancy specific factsheets on healthy eating, exercise and the benefits of healthy weight-gain during pregnancy and a general information booklet and diary for setting and tracking health goals. Women will receive usual care from their maternity clinicians during the trial with the exception to setting their weight-gain range target and general advice about gestational weight gain at their first antenatal visit with their midwife.

#### Information only arm

Women in the information only arm will receive a one off information and coaching session from a health coach and will receive the information materials described above (with exception of the diary). Women will receive usual care from their maternity clinicians during the trial with the exception of setting their weight-gain range target and general advice about gestational weight gain at their first antenatal visit with their midwife.

### Data collection

#### Quantitative

Data for the study will be collected at baseline (prior to 18 weeks gestation), 36 weeks gestation and 12 months post-birth. Baseline, endpoint and follow up data will be self-reported by the women. These data will include validated dietary [35–37] and physical activity [38, 39] measures. Baseline and endpoint data for the coaching arm will be collected by the health coach for the coaching arm and by a research assistant for the information only arm. Coaching participants who discontinue the coaching program without withdrawing from the study will be contacted by the research assistant for their endpoint data collection. The 12 months post birth data will be collected by the research assistant.

#### Qualitative interviews

Semi-structured telephone interviews (Additional files 1, 2 and 3) will be conducted by three researchers experienced in qualitative data collection methods (VC, KL and JR). Interviews will be recorded using a digital recorder and transcribed by a professional transcription service. Interview guides will be used to illicit the study participants’ experiences of the service, usefulness of program resources and suggestions for service improvement. Interviews will be conducted with the following:

- Up to 22 pregnant women in the coaching arm. A subset of this group will include women who actively withdraw from the coaching program.
- Up to 18 pregnant women in the information only arm.
- Up to 35 healthcare professionals (including clinic managers, midwives, doctors, and health coaches) who have contributed to the program by coordinating at the hospitals, recruiting pregnant women, providing medical clearance or delivering the service.

### Data analysis

#### Primary objectives

All quantitative data analysis will be based on intention to treat principles. Differences between the two study groups in self-reported weight change from pre-pregnancy weight to weight at 36 weeks gestation and 12 months post birth will be tested using the independent samples *t*-test. The proportions of pregnant women who achieve weight gain within the recommended range and those who return to their pre-pregnancy weight 12 months post birth will be analysed using the *X*^2^ test. The adjusted means of the BMI outcomes at 36 weeks gestation and follow up by BMI category will be compared with the pre pregnancy BMI.

Interview data will be analysed using the thematic analysis approach. The transcribed interviews will be coded by the researchers and if there is a disagreement in the classification of interview responses, the researchers will convene as a group to discuss the discrepancies and revise coding categories if needed. Once agreement is reached, themes and sub-themes will be extracted from the data. Data from the interviews will inform the provision of recommendations for program improvement.

#### Secondary objectives

Predictors of weight gain within the recommended range at 36 weeks of gestation will be analysed using multilevel linear mixed model. Covariance effects of hospital clusters and study arms will be incorporated into the models as random effects. Then the factors that contribute to BMI outcomes at 36 weeks will be determined with demographic variables, socio-economic status, age, pre pregnancy BMI and parity as the fixed effects.

Changes in dietary scores and confidence to change will be analysed using repeated measures analysis of variance, with the type of intervention and data collection point as between and within subject variables respectively.

Participation and retention in the service and preference for time and length of calls will be analysed using descriptive statistics with reference to the qualitative interview data where appropriate. The recommendations for service improvement will be one of the key themes in analysis of the qualitative data. The referral patterns of the hospitals will also be evaluated to assess recruitment rates with reference to the qualitative interviews with the midwives and doctors to identify the potential barriers and solutions to enhance service participation.

## Discussion

This study uses a robust design in a real-life routine practice setting to add additional knowledge and understanding to the growing body of evidence around interventions to target excessive gestational weight gain. This study is rare in that it considers both process and outcomes.

The pragmatic approach to the integration of the service with routine antenatal care will facilitate recruitment into the intervention in a clinical setting under realistic conditions without compromising program fidelity or requiring additional staff. Existing state-wide services will be utilised (routine antenatal care and the NSW Get Healthy Service) to recruit women to the service and provide the telephone-based coaching. The advantage of this approach is that the ‘real-world’ conditions of the trial will provide information to inform a rapid implementation of the service to other hospitals in NSW.

There are however unavoidable limitations to the pragmatic approach. Recruitment into GHiP may be influenced by local variables such as the strength of leadership, the presence of clinical champions, the individual approach of the midwives and organisation of care.

Integrating telephone-based health coaching with standard maternity care is a new concept for maternity services. The findings of this population-based study will help to determine if a telephone-based health coaching and information program is effective for women in supporting them to achieve a healthy gestational weight gain. In addition the trial seeks to examine the acceptability of the service to both women and maternity care providers.

## Additional files

Additional file 1: Coaching Participants - Topic Guide. (DOCX 556 kb) Additional file 2: Information Only Participants - Topic Guide. (DOCX 557 kb) Additional file 3: HPs - Topic Guide. (DOCX 556 kb)

## Abbreviations

BMI: Body mass index
EGWG: Excessive gestational weight gain
GHiP: Get Healthy in Pregnancy
GHS: Get Healthy Information and Coaching Service
IOM: Institute of Medicine
NSW: New South Wales
