# Supplementary material for: Pragmatic cluster randomised trial of a free telephone-based health coaching program to support women in managing weight gain during pregnancy: the Get Healthy in Pregnancy Trial
Source: BMC Health Serv Res. 2016 Aug 30;16(1):454. doi: 10.1186/s12913-016-1704-z (PMC5006383; doi:10.1186/s12913-016-1704-z)
Supplement: Additional file 1: — Coaching Participants - Topic Guide. (DOCX 556 kb) [file 12913_2016_1704_MOESM1_ESM.docx]

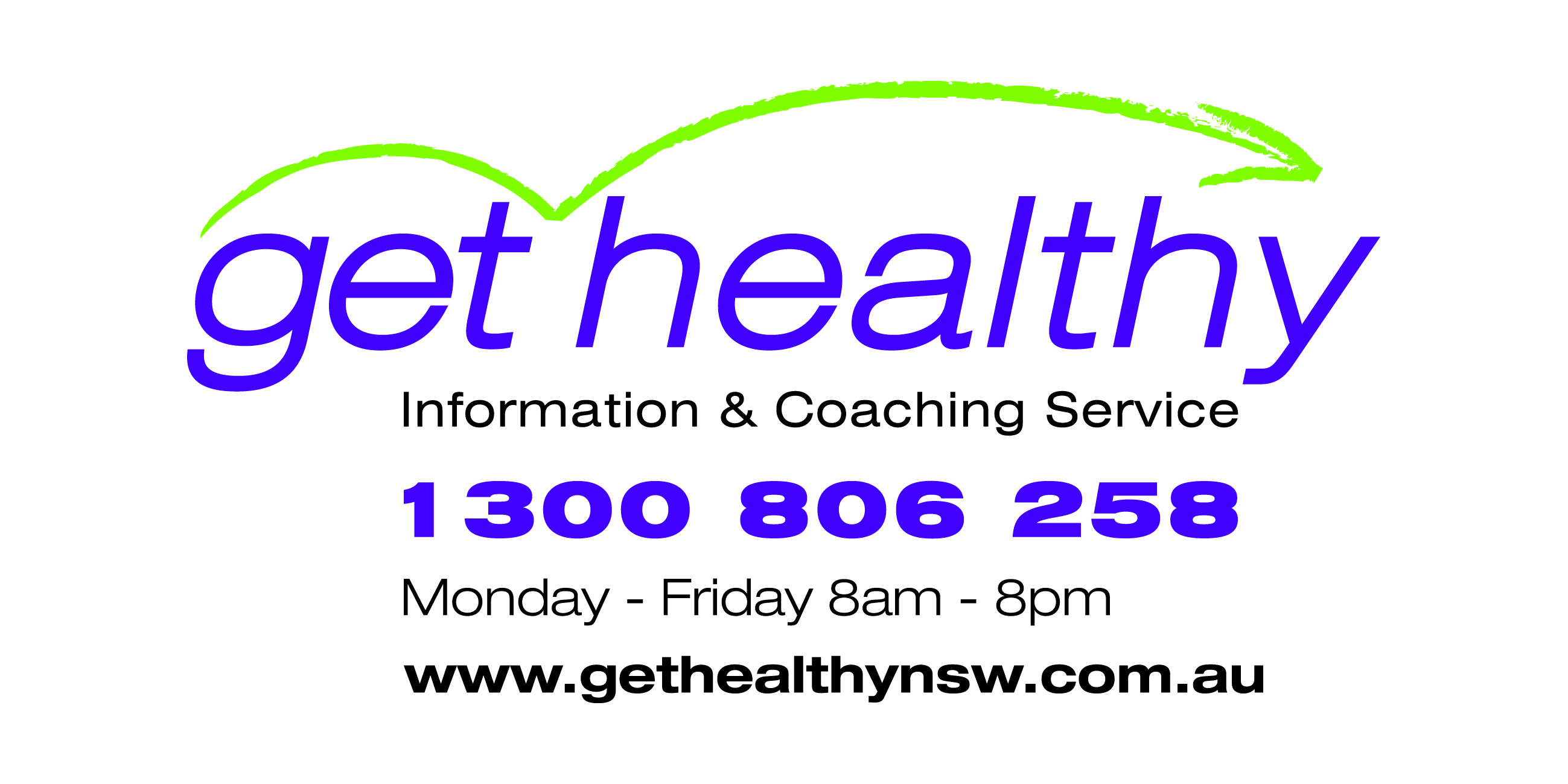


Topic Guide for Qualitative interviews with select coaching participants

**Evaluation of a telephone based information and coaching program to reduce excessive gestational weight gain amongst pregnant women**

1. Overall, how would you describe your experience on the coaching program?

*Probe: what elements of the coaching program did you find most helpful? (E.g. materials, quality of coaching)*

*What elements of the coaching program did you find least helpful? (E.g. materials, quality of coaching)*

1. Are there any changes you would recommend to the program to make it more appropriate for pregnant women?

*Probe: for example, was the frequency and/or length of calls right for you?*

1. if you withdrew from the coaching program prior to completion, what were the reasons for this?

*Probe: is there anything the Service could have offered to dissuade you from withdrawing?*
