# Supplementary material for: Pragmatic cluster randomised trial of a free telephone-based health coaching program to support women in managing weight gain during pregnancy: the Get Healthy in Pregnancy Trial
Source: BMC Health Serv Res. 2016 Aug 30;16(1):454. doi: 10.1186/s12913-016-1704-z (PMC5006383; doi:10.1186/s12913-016-1704-z)
Supplement: Additional file 2: — Information Only Participants - Topic Guide. (DOCX 557 kb) [file 12913_2016_1704_MOESM2_ESM.docx]

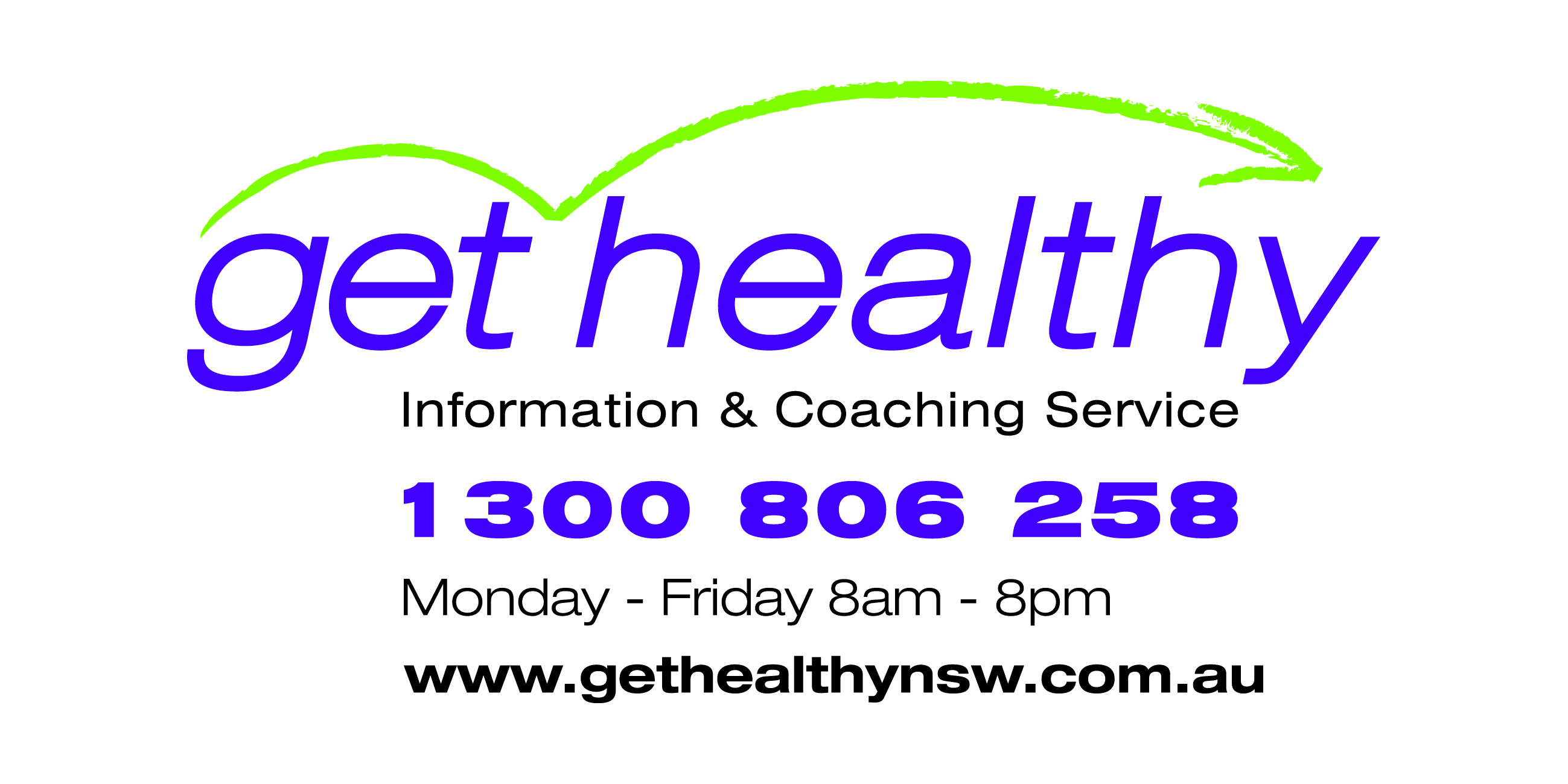


Topic Guide for Qualitative interviews with select information only participants

**Evaluation of a telephone based information and coaching program to reduce excessive gestational weight gain amongst pregnant women**

1. Overall, how would you describe your experience in the trial?

*Probe: What elements did you find most helpful? (E.g. materials, one off advice session)*

*Probe: What elements did you find least helpful? (E.g. materials, one off advice session)*

1. Are there any changes you would recommend to the materials to make them more appropriate for pregnant women?
