# Supplementary material for: Pragmatic cluster randomised trial of a free telephone-based health coaching program to support women in managing weight gain during pregnancy: the Get Healthy in Pregnancy Trial
Source: BMC Health Serv Res. 2016 Aug 30;16(1):454. doi: 10.1186/s12913-016-1704-z (PMC5006383; doi:10.1186/s12913-016-1704-z)
Supplement: Additional file 3: — HPs - Topic Guide. (DOCX 556 kb) [file 12913_2016_1704_MOESM3_ESM.docx]

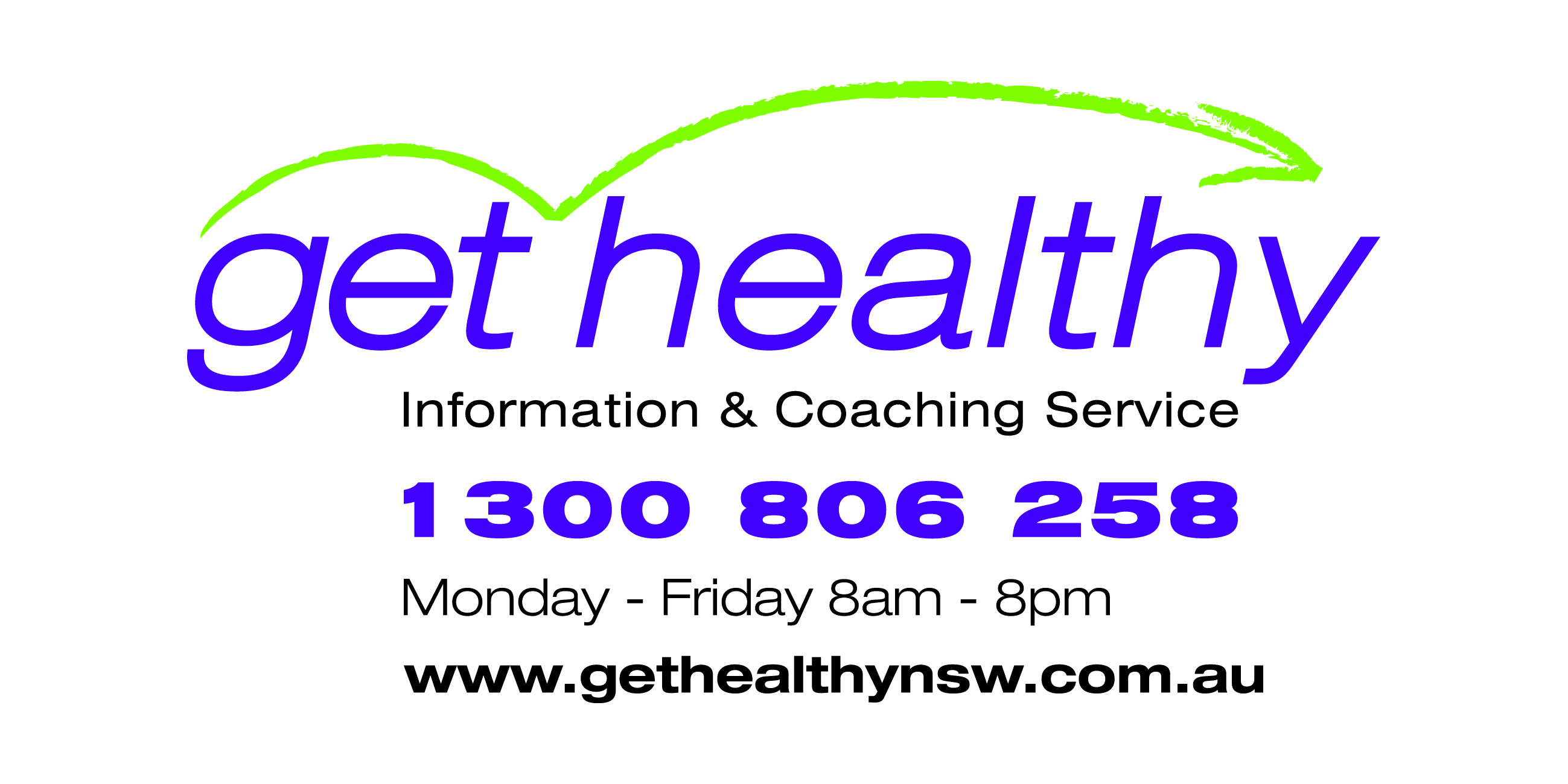


Topic Guide for Qualitative interviews with select midwives

**Evaluation of a telephone based information and coaching program to reduce excessive gestational weight gain amongst pregnant women**

1. Overall, how would you describe your experience referring women either into the coaching or information only group in this trial?

*Probe: what changes, if any, would you recommend to the referral process?*

1. What barriers, if any, exist for you in discussing and addressing healthy weight gain with pregnant clients?

*Probe: How might these barriers be addressed?*

*Probe: What other strategies exist to support healthy weight gain in pregnant clients?*

1. What additional resources or training, if any, would assist you in supporting pregnant clients to achieve healthy weight gain?
2. What ongoing collaboration, if any, would you like to see between midwives and GHS coaches in supporting pregnant women to achieve healthy weight gain?
